# The effects of case/control ratio and sample size on genome‐wide association studies: A simulation study

**DOI:** 10.1002/vms3.1444

**Published:** 2024-04-06

**Authors:** Ali Osman Turgut, Davut Koca

**Affiliations:** ^1^ Faculty of Veterinary Medicine Department of Animal Science Siirt University Siirt Turkey; ^2^ Faculty of Veterinary Medicine Department of Obstetrics and Gynecology Van Yüzüncü Yıl University Van Turkey

**Keywords:** case/control ratio, diseases, GWAS, sample size, simulation

## Abstract

**Background:**

Genome‐wide association studies (GWAS) is a useful tool for the detection of disease or quantitative trait‐related genetic variations in the veterinary field. For a binary trait, a case/control experiment is designed in GWAS. However, there is limited information on the optimal case/control and sample size in GWAS.

**Objectives:**

In this study, it was aimed to detect the effects of case/control ratio and sample size for GWAS using computer simulation under certain assumptions.

**Method:**

Using the PLINK software, we simulated three different disease scenarios. In scenario 1, we simulated 10 different case/control ratios with increasing ratio of cases to controls. In scenario 2, we did versa of scenario 1 with the increasing ratio of controls to cases. In scenarios 1 and 2, sample size gradually was increased with the change case/control ratios. In scenario 3, the total sample size was fixed to 2000 to see real effects of case/control ratio on the number of disease‐related single nucleotide polymorphisms (SNPs).

**Results:**

The results showed that the number of disease‐related SNPs were the highest when the case/control ratio is close to 1:1 in scenarios 1 and 2 and did not change with an increase in sample size. Similarly, the number of disease‐related SNPs was the highest in case/control ratios 1:1 in scenario 3. However, unbalanced case/control ratio caused the detection of lower number of disease‐related SNPs in scenario 3. The estimated average power of SNPs was highest when case/control ratio is 1:1 in all scenarios.

**Conclusions:**

All findings led to the conclusion that an increase in sample size may enhance the statistical power of GWAS when the number of cases is small. In addition, case/control ratio 1:1 may be the optimal ratio for GWAS. These findings may be valuable not only for veterinary field but also for human clinical experiments.

## Graphical abstract

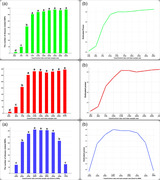

## 1 INTRODUCTION

Diseases cause important economic losses in animal breeding. Many diseases affect production traits such as milk, growth, meat, and reproductive traits in ruminants (Harris & Barletta, 2001; Ott et al., 1999). There are individual differences in resistance to diseases in a population. Mostly, these individual differences originate from genetic variations between animals (Alpay et al., 2014; Finlay et al., 2012; Kirkpatrick et al., 2022). On the other hand, some herd problems such as stillbirth, reproductive diseases, and infertility are thought to be related to multigenic factors (Jaureguiberry et al., 2020; Kasimanickam et al., 2020; Naderi et al., 2018; Wang et al., 2012). These herd problems cause important economic losses in animal breeding (Bage et al., 2002; Katagiri et al. 2013; Wang et al., 2012). Therefore, the detection of genetic variations associated with resistance to diseases or herd problems is an important subject. At this point, molecular genetic approaches offer opportunities. Genome‐wide association studies (GWAS) is a useful tool for the detection of genetic variations that may affect binary traits in ruminants (Alpay et al., 2014; Mastrangelo et al., 2019; Minozzi et al., 2022). To detect genetic variations related to a binary trait, case/control studies are designed in GWAS.

The optimal case–control ratio and the sample size are important subjects in GWAS. In human clinical experiments, the case–control ratio of 1:4 is accepted as a golden standard (Hong & Park, 2012). It is stated that the case–control ratio 1:4 is effective and increases the statistical power of single nucleotide polymorphism (SNP) association analysis (Kang et al., 2009). In the veterinary field, different case–control ratios and sample size are preferred for GWAS (Alpay et al., 2014; Kirkpatrick et al., 2022; Lei et al., 2021; Pausch et al., 2014). In addition, some GWAS experiments are carried out with small sample sizes because it is hard to obtain samples (Lei et al., 2021). Limited data exist on the effects of case/control ratio and sample size on GWAS (Hong & Park, 2012; Li et al., 2019; Moore et al., 2020). On the other hand, the optimal case/control ratio in GWAS is still unknown. Therefore, many researchers tend to disregard the significance of conducting statistical power assessments and determining the appropriate sample size in GWAS.

In this simulation study, we aimed to detect the optimal case/control ratio and sample size for GWAS under certain assumptions. We also aimed to show the effects of different case–control ratios and sample sizes on GWAS results using different case/control disease scenarios.

## 2 MATERIALS AND METHODS

### 2.1 Simulation

In the study, the single nucleotide polymorphism (SNP) dataset was generated using the PLINK software (v1.90) (Purcell et al., 2007) with the “–simulate” option. A total of 100,400 SNPs were generated for the disease. The case/control simulation for the disease included 100,000 null SNPs and 400 disease‐related SNPs. Frequency distribution of both “null” SNPs and disease‐related SNPs in the case/control group ranged between 0 and 1. The prevalence of the disease was set as 0.01. SNPs with minor allele frequency <0.05 were excluded from the analysis.

In scenario 1, we simulated 10 case/control ratios with increasing ratio of cases to controls; 1:10 (50/500, 550 total), 1:5 (100/500, 600 total), 1:2 (250/500, 750 total), 1:1 (500/500, 1000 total), 1.5:1 (750/500, 1250 total), 2:1 (1000/500, 1500 total), 3:1 (1500/500, 2000 total), 4:1 (2000/500, 2500 total), 5:1 (2500/500, 3000 total), and 10:1 (5000/500, 5500 total).

In scenario 2, we did versa of scenario 1; 10:1 (500/50), 5:1 (500/100), 2:1 (500/250), 1:1 (500/500), 1:1.5 (500/750), 1:2 (500/1000), 1:3 (500:1500), 1:4 (500:2000), 1:5 (500/2500), and 1:10 (500/5000).

In scenario 3, we fixed the total sample size to 2000 to see the real effects of case/control ratio on the number of disease‐related SNPs. For this purpose, we simulated nine different case/control ratios as 1:19 (100/1900), 1:7 (250/1750), 1:3 (500/1500), 1:1.66 (750/1250), 1:1 (1000/1000), 1.66:1 (1250/750), 3:1 (1500/500), 7:1 (1750/250), and 19:1 (1900/100).

### 2.2 Association analysis

In real GWAS studies, SNP quality control (QC) is performed on genomic data for more reliable results. However, in this study, we did not perform PLINK QC since the software generated a filtered SNP set already (100,400 SNPs). Association analysis was performed separately for each scenario using PLINK “–assoc” option. Bonferroni corrected *p*‐value was calculated using *α*/*n* formula, where *α* is the critical significant level (0.05) and *n* is the number of tests (SNPs). Bonferroni corrected *p*‐value was *p* < 5 × 10^−7^. All association analysis output files included the *p*‐value of each SNP for all case/control ratios. The basic association analysis of PLINK for case/control, chi‐square, was performed for association analysis.

To detect how many SNPs are related to disease, we carried out association analysis by filtering *p*‐values at *p* < 5 × 10^−7^ (Bonferroni corrected *p*‐value) for each case/control ratio using PLINK “–pfilter” option. Eventually, the number of SNPs related to the disease was detected. All output files that contain the number of disease‐related SNPs were used for statistical analysis.

### 2.3 Statistical analysis

Chi‐square analysis was performed to assess whether there is a significant difference between case/control ratios in case of the number of disease‐related SNPs. Statistical analysis was performed using Minitab (Version: 19.2020.2.0).

### 2.4 Power analysis

The estimated average power for disease‐related SNPs was calculated using the *S*/*m* formula, where *S* is the number of SNPs declared to be significant among disease SNPs and *m* is the disease‐related SNPs (Kang et al., 2009). The estimated average power was calculated for each case/control ratio.

## 3 RESULTS

### 3.1 Association analysis

Following PLINK association analysis, the number of disease‐related SNPs was detected by filtering *p*‐value at 5 × 10^−7^ for each scenario. The number of disease‐related SNPs was 9, 49, 208, 313, 320, 325, 334, 339, 334, and 340 in scenario 1.

In scenario 2, the number of disease‐related SNPs was 12, 51, 208, 315, 332, 332, 322, 330, 336, and 340.

In fixed sample size (in scenario 3), the number of disease‐related SNPs was 70, 260, 326, 360, 355, 355, 337, 268, and 75.

### 3.2 Statistical analysis

**FIGURE 1:**
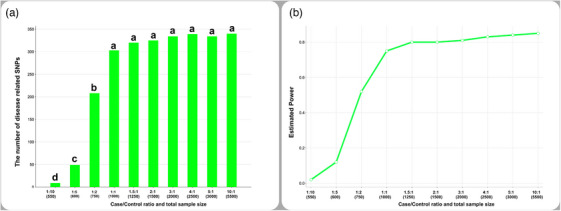
Bar charts showing changes in the number of disease‐related related single nucleotide polymorphisms (SNPs) (a) and estimated average power (b) in scenarios 1. Different letters (a–d) between case/control ratios show a statistically significant difference (*p* < 0.05).

The number of disease‐related SNPs was the lowest at the 1:10 (50/500) case/control ratio and increased gradually with the increasing ratio of cases to controls in scenario 1. However, the number of disease‐related SNPs did not change significantly following case/control ratio 1:1 (500/500) or higher case/control ratios (Figure 1a).

**FIGURE 2:**
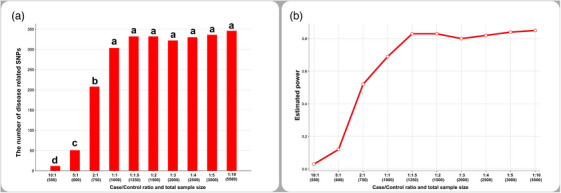
Bar charts showing changes in the number of disease‐related related single nucleotide polymorphisms (SNPs) (a) and estimated average power (b) in scenario 2. Different letters (a–d) between case/control ratios show a statistically significant difference (*p* < 0.05).

In scenario 2, the number of disease‐related SNPs was the lowest at the 10:1 (500/50) case/control ratio and increased gradually with the increasing ratio of controls to cases. However, the number of disease‐related SNPs did not change significantly between 1:1 or higher case/control ratios (Figure 2a).

**FIGURE 3:**
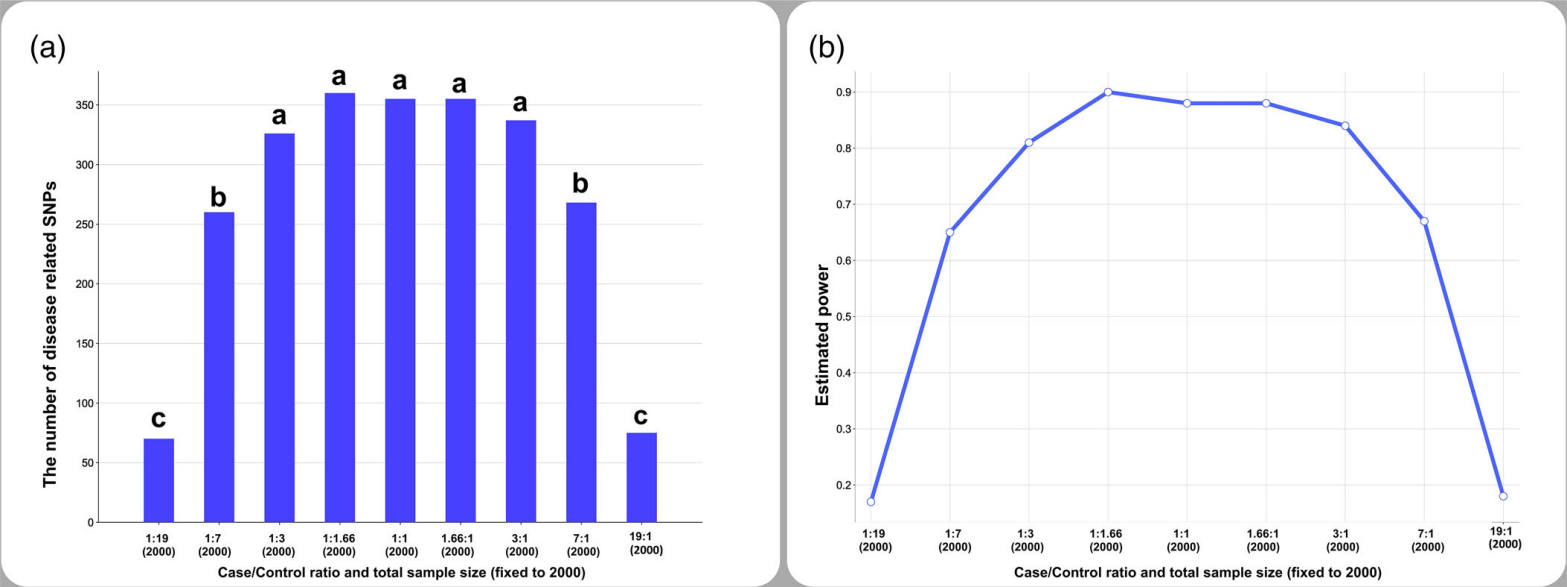
Bar charts showing changes in the number of disease‐related related single nucleotide polymorphisms (SNPs) (a) and estimated average power (b) in scenario 3. Different letters (a–d) between case/control ratios show a statistically significant difference (*p* < 0.05).

In scenario 3, the number of disease‐related SNPs gradually increased with the rise of cases to controls, reached the highest number when the case/control ratio is close to 1:1, and decreased thereafter. The number of disease‐related SNPs was the lowest at 1:19 (100/1900) and 19:1 (1900/100) case/control ratios (Figure 3a).

### 3.3 Power analysis

In scenario 1, the estimated average power for disease‐related SNPs for 1:10, 1:5, 1:2, 1:1, 1.5:1, 2:1, 3:1, 4:1, 5:1, and 10:1 case/control ratios was 0.03, 0.12, 0.52, 0.75, 0.80, 0.81, 0.83, 0.84, 0.83, and 0.85, respectively (Figure 1b).

In scenario 2, the estimated average power for disease‐related SNPs for 10:1, 5:1, 2:1, 1:1, 1:1.5, 1:2, 1:3, 1:4, 1:5, and 1:10 case/control ratios was 0.03, 0.12, 0.52, 0.69, 0.83, 0.83, 0.80, 0.82, 0.84, and 0.85, respectively (Figure 2b).

In fixed sample size, the estimated power for disease‐related SNPs for 100/1900, 250/1750, 500/1500, 750/1250, 1000/1000, 1250/750, 1500/500, 1750/250, and 1900/100 case/control ratios was 0.17, 0.65, 0.81, 0.90, 0.88, 0.88, 0.84, 0.67, and 0.18, respectively (Figure 3b).

## 4 DISCUSSION

In a real GWAS analysis, thousands of SNPs are tested. Therefore, different statistical approaches such as false discovery rate, Sidak correction, Bonferroni correction, and Bayesian approaches have been proposed for GWAS analysis. One of the most common approaches used for GWAS analysis is the Bonferroni correction. This method is accepted as the most conservative approach because it assumes that each SNP is independent of the rest of the SNPs (Kaler & Purcell, 2019). Bonferroni's correction reduces the probability of a type I error (false positive) occurring during multiple testing (Sedgwick, 2014). Therefore, we carried out association analyses using Bonferroni correction (*p* < 5 × 10^−7^).

In human clinical research, the 1:4 case/control ratio is accepted as a golden standard (Hong & Park, 2012), and it was reported that statistical power increased with the increase in controls to cases. In a study, Kang et al. (2009) simulated 50 disease‐related and 300 non‐disease SNPs for 50/50 (1:1), 50/100 (1:2), 50/150 (1:3), and 50/200 (1:4) case/control ratios. They found that an increase in controls to cases enabled the detection of more disease‐related SNPs. In addition, they reported that an increase in the ratio of controls to cases positively affects statistical power at the *p* < 0.05 threshold. Similarly, we have detected that an increase in ratio of controls to cases enables to detect significantly more disease‐related SNPs in scenario 2 (Figure 2b). Furthermore, we also detected that increasing the ratio of the cases to control resulted in the detection of more disease‐related SNPs interestingly (Figure 2a). However, the number of disease‐related SNPs did not change significantly after 1:1 (500/500) case/control ratio for scenarios 1 and 2. These findings show that an increase in sample size enables to detect more disease‐related SNPs. On the other hand, detectable disease‐related SNPs reach peak level when case/control ratio is 1:1 for this simulation study. Therefore, the detection of more disease‐related SNPs in scenarios 1 and 2 may be due to the increase in total sample size.

In scenario 3, we fixed the total sample size to 2000 to see the real effect of the case/control ratio on the GWAS result. After analysis, we found that the number of disease‐related SNPs was higher when the case/control ratio is 1:1. Furthermore, unbalanced case/control ratios have resulted in the detection of the lower number of disease‐related SNPs in scenario 3. Therefore, it seems that the optimal case/control ratio may be 1:1 for GWAS.

The sample size is an important determinant for case/control studies. Akobeng (2016) reported that small sample size for case/control studies may cause unreliable results. In addition, he suggested that the results for small sample sizes need to be assessed carefully and that the statistical power of the test should be calculated. In this study, we also simulated small case/control ratios such as 50/50, 50/100, 50/150, and 50/200 using the scenario of Kang et al. (2009) for 100,400 SNPs and carried out association analysis. However, the number of disease‐related SNPs was too low compared to the bigger sample size in this study (data not shown). These findings led to the conclusion that the total sample size may affect GWAS results and confirm the reports of Akobeng (2016).

In this study, we calculated the average power for each case/control scenario using a formula described by Kang et al. (2009). Because an increase in total sample size enabled the detection of more disease‐related SNPs, statistical power increased gradually in scenarios 1 and 2. In the case/control studies, 80% statistical power is the minimum acceptable power (Akobeng, 2016; Sedgwick, 2013). In this study, the estimated average power for SNPs was 80% or higher when the case/control ratio is 1:1 and did not change significantly in the higher case/control ratios in scenarios 1 and 2. However, in scenario 3, the estimated average power decreased with the unbalanced case/control ratios (Figure 3b). Moore et al. (2020) reported that the statistical power of the GWAS is higher when case/control ratio is 1:1. Similarly, Hong and Park (2012) also showed that the statistical power of the test reaches 80% when case/control ratio is 1:1 in accordance with this study. Therefore, we concluded that the optimal case/control ratio with acceptable statistical power may be 1:1 in GWAS design with an appropriate sample size.

In the veterinary field, GWAS is commonly used for the detection of genetic variations related to a trait. In this respect, GWAS is used for the detection of SNPs related to resistance to diseases such as *Mycobacterium Avium* subspecies *paratuberculosis *(Alpay et al., 2014; Canive et al., 2021; Fisher et al., 2011; Kirkpatrick et al., 2022; Minozzi et al., 2022), bovine leukemia virus (Brym et al., 2016; Carignano et al., 2018), bovine spongiform encephalopathy (Murdoch et al., 2010), and ovine foot‐root disease (Raadsma et al., 2018). In addition, cattle idiopathic male subfertility (Pausch et al., 2014) and coat colour‐related genetic variations (Mastrangelo et al., 2019) are evaluated by GWAS. In these studies, a different case–control ratio and a different sample size are preferred. Some researchers prefer a case/control ratio of 1:1 (Alpay et al., 2014; Canive et al., 2021; Minozzi et al., 2022), while others prefer a case/control ratio of 1:4 (Fisher et al., 2011; Kirkpatrick et al., 2022). Furthermore, association analysis is carried out using unbalanced case/control ratio in some studies (Pausch et al., 2014). According to our findings and previous reports, it is obvious that unbalanced case–control ratios and small sample size may decrease statistical power and affect the GWAS results negatively. Therefore, taking into account case/control balance may be beneficial for the reliability and efficiency of the GWAS. In addition, because detectable SNP counts reach a peak level in big sample size, increasing the sample size may not be cost‐efficient for GWAS. GWAS simulations with generated or real data may be useful to predict required sample size.

## 5 CONCLUSION

In summary, we have shown that case/control ratio and sample size can affect GWAS results and statistical power. Increasing the sample size may enhance the statistical power of GWAS when the number of cases is small. Furthermore, the case/control ratio 1:1 may be the optimal ratio and may increase the statistical power of the test in GWAS. These findings may be valuable not only for veterinary field but also for human clinical experiments.

## AUTHOR CONTRIBUTIONS

Ali Osman Turgut: Conceptualization, writing‐original draft, visualization, software, investigation, review and editing; Davut Koca: Conceptualization, writing‐original draft, visualization, investigation, review and editing

## CONFLICT OF INTEREST STATEMENT

The authors declare no conflicts of interest.

## FUNDING INFORMATION

No institution or organization supported this study.

## ETHICS STATEMENT

Ethical approval is not needed because this is a simulation study.

## Data Availability

The data that support the findings of this study are available from the corresponding author upon reasonable request.
